# Pain and fatigue in adult patients with multiple osteochondromas: The Netherlands

**DOI:** 10.1371/journal.pone.0305640

**Published:** 2024-07-17

**Authors:** Ihsane Amajjar, Kuni Vergauwen, Nienke W. Willigenburg, S. John Ham, Rob J. E. M. Smeets

**Affiliations:** 1 Department of Orthopaedic Surgery, OLVG, Amsterdam, The Netherlands; 2 Department of Rehabilitation Medicine, CAPHRI, Maastricht University, Maastricht, The Netherlands; 3 Department of Health Care, AP University College, Antwerp, Belgium; 4 MOVANT, Department of Rehabilitation Sciences and Physiotherapy, University of Antwerp, Antwerp, Belgium; 5 Pain in Motion International Research Group, Brussels, Belgium; 6 Clinics in Rehabilitation, Eindhoven, The Netherlands; Zarqa University, JORDAN

## Abstract

**Background:**

Multiple Osteochondromas (MO) is a rare genetic disorder characterised by the presence of numerous benign bone tumours, known as osteochondromas. Within the spectrum of debilitating symptoms associated with MO, pain is recognized as a major problem. Interestingly, our clinical observations suggest that fatigue is also a significant concern but has merely been touched upon in MO literature. This study aims to (1) assess the level of pain and fatigue in adult patients with MO; (2) compare fatigue in MO to healthy subjects and patients with Rheumatoid Arthritis (RA); (3) identify associated variables for pain and fatigue in patients with MO.

**Methods:**

In this cross-sectional study, 353 adult MO patients completed a survey with validated questionnaires on pain, fatigue and psychosocial factors. Pain and fatigue were assessed with the Numeric Rating Scale (NRS), and fatigue was also measured with the Checklist Individual Strength (CIS). Fatigue (CIS) was compared with reference scores of healthy subjects and patients with RA, using a one-sample t-test. Multiple linear regression models for pain and fatigue were developed using a-priori selected independent variables based on a theoretical framework (ICF-model).

**Results:**

Pain was reported by 87.8% (NRS = 3.19±2.6) and fatigue by 90.4% (NRS = 4.1±2.6) of patients with MO. Fatigue scores for MO (CIS = 84.1±15.3) were significantly higher (p<0.001) compared to reference scores of healthy subjects and patients with RA. The multivariable analysis for pain provided a final regression model with six variables (R^2^ = 0.445, p<0.001) of which fear avoidance beliefs and fatigue had the strongest association. For the fatigue models NRS (R^2^ = 0.455, p<0.001) and CIS (R^2^ = 0.233, p<0.001), the strongest associations were found with anxiety and depression respectively.

**Conclusions:**

Pain and fatigue are highly prevalent in patients with MO. Fatigue is significantly higher compared to healthy subjects and patients with RA. Several variables associated with pain and fatigue have been identified that could help improve multidisciplinary treatment plans.

## Introduction

Multiple Osteochondromas (MO) is a rare genetic disorder characterized by the presence of numerous bony outgrowths covered by a cartilage cap that develop at the metaphysis of long bones [1]. These outgrowths, also called osteochondromas, can cause severe complications such as compression of nerves, vessels, tendons and muscles, limb deformities, restriction in mobility, and in 2–6% transform into chondrosarcomas [2–5]. As a result of these MO related complications surgery is performed in 66%-88% of the patients [6–8]. Mutations in EXT-1 and EXT-2 on chromosome 8 and 11, respectively, are found in 90% of the European patients with MO [9]. Some studies report more severe symptoms for EXT-1 mutations [5,10,11] and male gender [2,12], while others have not found a difference in disease severity based on mutation or gender [13,14]. Unfortunately, as there is still no medical treatment for MO, therefore the focus in healthcare is on symptom alleviation.

Previous literature reported that pain is one of the biggest problems in MO [6,7,15]. In our clinical practice, the national expertise centre for MO and rehabilitation clinic, fatigue seems to be a major problem as well. Fatigue has not received much attention in literature, nor in clinical care of patients with MO. To our knowledge, only the study of Bathen et al. (2019) reported on fatigue in MO. In their small series of 21 adult patients, significantly higher fatigue scores were reported compared to the general population [16]. The patients were recruited from a national registry of rare diseases and the Norwegian MO association. However, due to the small study group and possible selection bias this data cannot be generalized to the whole MO population. Further research is warranted to examine the exact impact of fatigue in MO and possible associating factors such as pain.

Several studies in other chronic diseases have reported on the co-occurrence of pain and fatigue [17,18]. In the general Dutch population, Creavin et al. found an association of both pain and fatigue with high BMI, low levels of activity, chronic disease, anxiety and depression [19]. Both pain and fatigue were also associated with increased healthcare usage [20]. It is known that fatigue does not resolve spontaneously and can be a significant predictor of work and activity impairment in several chronic diseases [21,22]. Previous studies on pain and quality of life in the MO-population reported that 26% to 49% of patients experienced job-related issues due to pain [7,15]. However, these studies did not collect data on the level and impact of fatigue, or its potential associated effects.

The objectives of the present study are: (1) to assess the level of pain and fatigue in male and female patients with MO; (2) to compare the prevalence and severity of fatigue in MO to gender matched healthy subjects and patients with rheumatoid arthritis (RA); and 3) to identify associated variables for pain and fatigue in patients with MO. The ultimate goal is to improve treatment strategies for patients with MO and potentially even prevent pain, fatigue, and their subsequent problems in this population.

**Fig 1:**
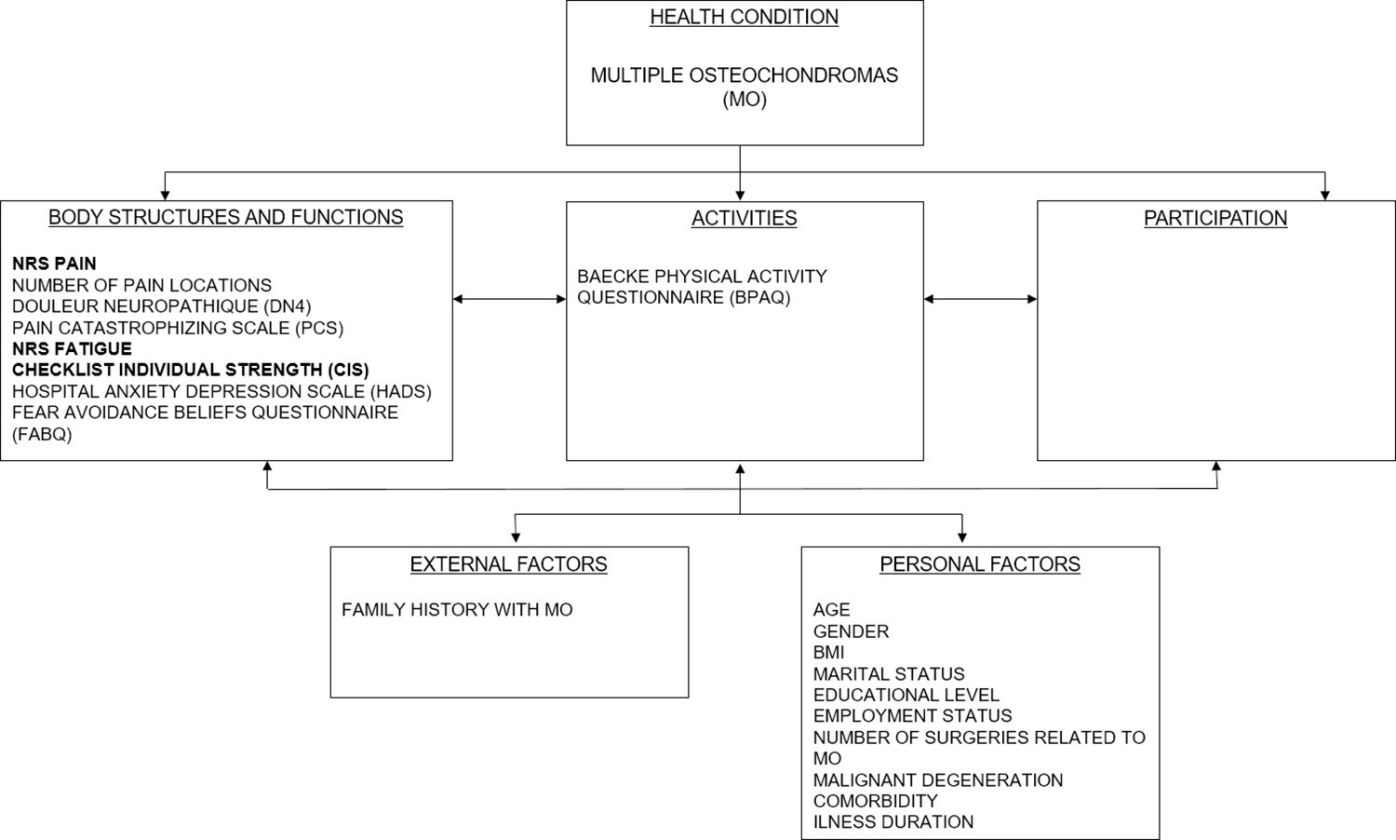
ICF model with dependent and possible associated variables for pain and fatigue. In bold the measures for the dependent variables.

We used The International Classification of Functioning, Disability and Health (ICF) model [23] (Fig 1) as theoretical framework. Based on our study aims, clinical reasoning, and previous literature on MO, we formulated the following hypotheses: (1) patients with MO experience more pain and fatigue than healthy controls and patients with RA; (2) Pain in MO is independently associated with fatigue; (3) Pain and fatigue scores are negatively associated with the presence of psychological factors and level of physical activity; (4) a higher BMI, older age, male gender, a lower educational level, unemployment, and being single (marital status) are negatively associated with pain and fatigue; (5) the amount of surgical procedures, the amount of pain location sites, a negative family history for MO, comorbidities and malignant degeneration into chondrosarcoma are also negatively associated with pain and fatigue.

## Methods

### Study design and participants

A cross-sectional survey study was performed at the Dutch national MO expertise centre (OLVG, Amsterdam) in collaboration with the patient association ‘HME-MO Vereniging Nederland’. Patients 18 years and older diagnosed with MO were recruited between May 2018 and January 2020 during their outpatient clinic visit or contacted by telephone. The Dutch patient association also posted an open invitation on their website and newsletter about this survey study. Individuals under the age of 18, those without a diagnosis of MO, and those not proficient in Dutch were excluded from the study to ensure accurate comprehension of the survey questions. After informed consent was signed, participants received a comprehensive survey including questions on sociodemographics and several validated questionnaires on pain, fatigue, anxiety and depression.

### Data collection

Data was collected using the electronic data capture system, Castor EDC [24]. The Castor EDC software reduced the risk of missing data by preventing patients to skip questions. In case of missing data for patients who received a paper survey, the coordinating researcher reached out and in case there were still missing data these were handled according to the questionnaire’s specific instructions. The main outcome parameters, pain and fatigue, were measured with several questionnaires outlined below. The other parameters, i.e., possibly associated (independent) variables, were selected beforehand based on evidence from existing literature and a-priori formed hypotheses based on the ICF-model (Fig 1).

### Dependent variables

Pain–Pain was measured using the Numeric Rating Scale (NRS; range 0–10, where 0 represents no pain and 10 indicates the worst imaginable pain). Patients were queried about the average severity of their pain over the past week.

Fatigue–Fatigue severity was also measured using the NRS (range 0–10, as mentioned above) and the validated questionnaire Checklist Individual Strength (CIS-total; range 20–140) [25]. The CIS-total consists of 20 items with 4 different aspects of fatigue: 1. Fatigue severity 2. Problems with concentrating 3. Motivation 4. Physical activity level. Fatigue severity was measured using the CIS-Subjective Fatigue subscale (8–56), with scores ≥ 35 indicating severe fatigue. For the total CIS score the cut-off was set at 76, indicating problematic fatigue [25,26].

### Independent variables (possible associated variables for pain and fatigue)

- Sociodemographic- and disease related factors as listed in Table 1.
- Neuropathic pain: The Douleur Neuropathique (DN4) is a validated questionnaire assessing pain characteristics and symptoms of abnormal sensations [27]. The DN4 consist of 2 parts, the questionnaire and the physical exam. For this study only the questionnaire was used.
- The Pain Catastrophizing Scale (PCS; range 0–52) was used to assess pain catastrophizing and to evaluate the emotional response to pain [28].
- The Fear Avoidance Beliefs Questionnaire (FABQ; range 0–96) was used to assess how patients’ fear avoidance beliefs about physical activity and work influence their pain [29].
- The Hospital Anxiety Depression Scale is a 14-item self-report that measures anxiety (7 items) and depression symptoms (7 items) [30].
- The Baecke Physical Activity Questionnaire (BPAQ; range 3–15) was used to evaluate physical activity levels over the previous 12 months [31]. The BPAQ consist of three domains: work, sport and leisure time.

**Table 1:** Patient characteristics, clinical data and independent variables.

	Total n = 353	Men n = 152	Women n = 201	p-value
**Age ± SD (years)**	41.6 ± 16.2 (17–91)	41.3 ± 16.4 (17–85)	41.9 ± 16.2 (18–91)	0.747
**Educational level %**				0.981
**Primary**	0.6	0.7	0.5	
**Secondary**	56.4	55.9	56.7	
**Tertiary**	40.8	41.4	40.3	
**Other**	2.3	2.0	2.5	
**Paid work (%)**	67.4	70.4	65.2	0.300
**Marital Status (%)**				0.463
**Single**	14.7	15.1	14.4	
**In a relationship**	79.3	81.6	77.6	
**Divorced**	3.7	2.0	5.0	
**Widowed**	2.3	1.3	3.0	
**Number of surgical procedures ± SD (range)**	6.8 ± 7.1 (0–55)	6.78 ± 7.58 (0–55)	6.85 ± 6.73 (0–36)	0.418
**0–2 surgeries (%)**	31.4	34.9	28.9	
**3–5 surgeries (%)**	24.1	20.4	26.9	
**6–10 surgeries (%)**	22.1	21.1	22.9	
**>10 surgeries (%)**	22.4	23.7	21.4	
**Comorbidity (%)**	24.1	17.8	28.9	0.016 ^**a**^
**Use of painkillers**	76	19	57	
**Paracetamol**	40	8	32	
**NSAIDs**	39	11	28	
**Opioids**	34	9	25	
**Neuropathic analgesics**	10	2	8	
**BMI ± SD**	26 ±5.2 (16.2–44.5)	26 ± 4.7 (17.7–44.1)	26.1 ± 5.5 (16.2–44.5)	0.858
**Family history of MO (%)**	81.0	79.6	82.1	0.556
**Malignant degeneration (%)**	8.5	10.5	7.0	0.235
**Disease duration ± SD (years)**	31.6 ± 17.1 (0–81)	30.4 ± 18 (0–81)	32.5 ± 16.4 (0–81)	0.244
**Amount of pain locations (%) ^b^**				<0.001^**a**^
***0***	12.2	18.4	7.5	
***1–2***	23.8	32.2	17.4	
***3–4***	21.2	19.7	22.4	
***5–7***	16.7	15.8	17.4	
***>7***	26.1	13.8	35.3	
**Positive DN4 (%) ^b^**	28.6	22.4	33.3	0.024 ^**a**^
**Pain Catastrophizing Scale ^b^**	12.7 ± 9.4 (0–46)	11.6 ± 9.3 (0–46)	13.6 ± 9.4 (0–42)	0.049 ^**a**^
**Fear Avoidance Beliefs Questionnaire ^b^**	31.3 ± 20.5 (0–92)	29 ± 21.5 (0–92)	33.2 ± 19.5 (0–84)	0.056
**HADS anxiety ^b^**	5.7 ± 3.9 (0–20)	4.8 ± 3.6 (0–18)	6.4 ± 4.0 (0–20)	<0.001 ^**a**^
**HADS depression ^b^**	3.7 ± 3.4 (0–20)	3.6 ± 3.6 (0–20)	3.8 ± 3.3 (0–18)	0.625
**Baecke Physical Activity Questionnaire—total score ^b^**	7.7 ± 1.4 (4.4–11.3)	7.6 ± 1.4 (4.4–11.3)	7.7 ± 1.3 (4.6–10.6)	0.497
***Work***	2.6 ± 0.7 (1–4.8)	2.5 ± 0.7 (1–4.8)	2.7 ± 0.6 (1.3–4.3)	0.088
***Sport***	2.4 ± 0.8 (1–4.3)	2.4 ± 0.6 (1–4.3)	2.2 ± 0.7 (1–4.0)	0.136
***Leisure time***	2.8 ± 0.7 (1.25–4.5)	2.7 ± 0.7 (1.3–4.5)	2.8 ± 0.8 (1.3–4.5)	0.486

^a^: p <0.05; Chi-square or independent samples T-test between men and women.

^b^: Independent variables.

Abbreviations: DN4, Douleur Neuropathic en 4 questions; HADS, Hospital Anxiety and Depression Scale.

### Statistical methods

All analyses were performed in SPSS (version 27 for Windows SPSS for Windows, Chicago, IL, USA). No power analysis was performed. We aimed for a sample size of at least 300 patients, based on the study of Goud and colleagues performed in the Dutch population [7]. The exact prevalence of MO in the Netherlands is unknown, but literature describes a prevalence rate of 1:50.000 in Western Countries [1,8]. This suggests at least 340 cases in the Netherlands’17milion population. Goud and colleagues, indicate the actual prevalence is probably higher given the number of patients included in their study (184 adults and 99 children) [7]. We aimed for a sample size of at least 300 adult patients, ensuring a substantial number of observations to enhance the accuracy of establishing statistical associations.

Descriptive data were reported for patient characteristics and the prevalence rates of pain, fatigue and the psychosocial variables. Gender differences were analysed using Chi-square or independent samples T-test. A one sample t- test was used to compare fatigue scores (CIS) to reference scores from both healthy subjects and patients with RA [25,32].

A purposeful selection method with 2 steps was used to develop three linear regression models. The dependent variables were NRS pain, NRS Fatigue, and CIS total, all of which are continuous variables. Step 1: the possible associated independent variables for fatigue and pain that were selected beforehand with the ICF models were analysed with univariable regression; those with a P value ≤ 0.25 were carried forward to the second step of the analysis. Step 2: the remaining independent variables were included in a backward multiple regression analysis with the significance level set at p ≤ 0.05 [33]. The independent variables were controlled for multicollinearity in the final model (Variation Inflation Factor, VIF<4 and Tolerance>0.1). The final models were checked for normality of residuals and homoscedasticity.

### Ethical approval

The study was approved by the Institutional Review Board ACWO-MEC (OLVG, Reference No. WO 17.067). Written informed consent was obtained. All participant data were anonymized and securely managed using Castor EDC. There were no risks associated with participation and participants could withdraw from the study at any time.

## Results

### Patient characteristics

A total of 392 adult patients were invited and 353 patients completed the survey (response rate: 90.1%). Of the 39 patients that did not fill in the survey after initial informed consent the most common reason of withdrawal was lack of time or loss of interest. Patients’ characteristics are outlined in Table 1. The previously mentioned gender differences in literature were also analysed in our study. Female patients reported significantly more comorbidities than male patients with MO (p = 0.016).

### Prevalence rates of pain, fatigue and independent variables in MO

The prevalence rates of pain, fatigue and independent variables are shown in Tables 1 and 2. Pain was reported by 87.8% of the patients with a mean NRS of 3.19 ±2.6. Male patients had significant lower pain scores than female patients (resp. mean NRS 2.5 vs 3.7). Neuropathic pain analysed with the DN4 questionnaire was more often described in female patients (resp. 22.4% vs. 33.3%, p = 0.024) and they also had a higher amount of pain location sites (p<0.001).

**Table 2:** Prevalence rates of fatigue and pain.

	Total n = 353	Men n = 152	Women n = 201	p-value
**NRS Pain ^a^**	3.19 ± 2.6 (0–10)	2.5 ± 2.4 (0–8)	3.7 ± 2.6 (0–10)	<0.001
**NRS Fatigue ^a^**	4.1 ± 2.6 (0–10)	3.3 ± 2.5 (0–10)	4.7 ± 2.5 (0–10)	<0.001
**CIS total ^a^**	84.1 ± 15.3 (35–123)	82.0 ± 16.1 (35–123)	85.7 ± 14.6 (49–122)	0.026
**CIS subjective fatigue ^a^**	36.2 ± 8.0 (15–52)	34.7 ± 8.5 (15–51)	37.3 ± 7.5 (18–52)	0.003
**CIS severe fatigue, cut off subj fatigue ≥35 (%)**	55.0	49.3	59.2	0.065

^a^: p <0.05; Chi-square or independent samples T-test between men and women.

Abbreviations: NRS, Numeric Rating Scale; CIS, Checklist Individual Strength.

Score interpretation: NRS ranges 0–10, with 0 indicating absence of pain/fatigue and 10 representing maximal pain/fatigue. For the CIS, elevated scores correspond to increased fatigue levels.

Fatigue was reported by 90.4% of the patients, mean NRS 4.1 ± 2.6, which was also significantly higher for women. Problematic fatigue, defined by a total score of >76 on the CIS-total questionnaire, was reported by 71,1%. Severe fatigue (CIS-Subjective Fatigue cut-off score ≥35) was present in 55% of the cases, without any significant gender differences.

Furthermore, female patients scored worse on anxiety and pain catastrophizing scales (resp. p = <0.0001, p = 0.049).

### Comparison of prevalence rates of fatigue to control groups

Patients with MO showed significantly higher fatigue scores compared to reference scores of healthy subjects from the general Dutch population[25], and patients with RA (p = <0.0001) [32]. The differences are outlined in Table 3.

**Table 3:** Comparison of means (T-test) for CIS total and CIS subjective fatigue (CIS-F) between MO patients and healthy controls and RA.

Variable	Comparison group	Mean Difference	95% CI	p
**CIS total MO**				
	Healthy controls ^a^	29.28	27.67; 30.88	p<0.0001
	Rheumatoid Arthritis ^b^	15.95	26.49; 23.28	p<0.0001
**CIS-F MO**				
	Healthy controls ^a^	13.20	12.36; 14.03	p<0.0001
	Rheumatoid Arthritis ^b^	4.54	3.70; 5.37	p<0.0001

^a^ Reference scores from Vercoulen et al., 1994 (The Netherlands) [25].

^b^ Reference scores from Hoogmoed et al., 2010 (The Netherlands) [32].

Abbreviations: CIS, Checklist Individual strength; CIS-F, Checklist Individual strength- Subjective Fatigue; MO, Multiple Osteochondromas.

### The relationship of pain and fatigue and other variables

The univariate analysis of pain and fatigue with the purposeful selected independent variables from the ICF models (Fig 1) can be found in S1 Appendix.

#### NRS Pain regression model

The final multiple regression model of pain for patients with MO is presented in Table 4. A total of six variables remained in the final model, together explaining 44.5% of the total variance.

**Table 4:** Summary of backward multiple linear regression analysis for pain (NRS) as dependent variable.

Independent variable	Unstandardized ß	R^2^	F	P- value	sr^2^	VIF
		.445	36.382	< .001		
**NRS fatigue**	.197			.000	0.031	1.422
**Fear Avoidance Beliefs Questionnaire**	.035			.000	0.054	1.349
**DN4+**	.982			.001	0.026	1.198
**Pain Catastrophizing Scale**	.056			.000	0.031	1.383
**>6–10 surgeries**	.810			.005	0.016	1.098
**>10 surgeries**	.741			.014	0.013	1.173

R^2^ = adjusted explained variance, sr^2^ = squared semi-partial correlation, VIF = variance inflation factor.

Abbreviations: NRS, Numeric Rating Scale; DN4, Douleur Neuropathic en 4 questions.

Pain was positively associated with fatigue and with higher number of surgical procedures. Also, the more patients catastrophize about their pain and show fear avoidance behaviour, the more pain is reported. When neuropathic pain was present, an increment of almost 1 point on the NRS scale for pain occurred. Fear avoidance beliefs explained the most unique variance (5.4%).

#### NRS fatigue regression model

Table 5 presents the final regression model for fatigue assessed with the Numeric Rating Scale (NRS). Eight variables remained in the final model, explaining 45.5% of the total variance. Living together was the only factor associated with less fatigue. Pain, depression, anxiety and fear avoidance beliefs were associated with higher fatigue scores. In case of more than 4 pain locations, the fatigue score increased with at least 1.3 points. Anxiety explained the most unique variance (6.0%).

**Table 5:** Summary of backward multiple linear regression analysis for fatigue NRS as dependent variable.

Independent variable	Unstandardized ß	R^2^	F	P- value	sr^2^	VIF
		0.455	28.629	< .0001		
**NRS pain**	0.147			0.031	0.010	1.955
**Pain locations:**						
**3–4**	0.815			0.015	0.012	1.374
**5–7**	1.373			0.001	0.025	1.571
**>7**	1.298			0.001	0.022	2.097
**Living together**	-0.715			0.030	0.010	1.024
**Anxiety HADS**	0.216			0.000	0.060	1.747
**Depression HADS**	0.100			0.049	0.008	1.891
**Fear Avoidance Beliefs Questionnaire**	0.017			0.029	0.010	1.481

R^2^ = adjusted explained variance, sr^2^ = squared semi-partial correlation, VIF = variance inflation factor.

Abbreviations: NRS, Numeric Rating Scale; HADS, Hospital Anxiety and Depression Scale.

#### CIS fatigue regression model

A second regression model for fatigue was assessed using the CIS total score as dependent variable (Table 6.). Only three variables remained in this model, explaining 23.3% of the total variance. Patients with a paid job showed fatigue scores that are 5.5 points lower. Pain and depression were associated with higher level of fatigue, and depression explained the highest unique variance (9%). In this model pain explained a unique variance of 2.8%.

**Table 6:** Summary of backward multiple linear regression analysis for fatigue using CIS-total scores as dependent variable.

Independent variable	Unstandardized ß	R^2^	F	P- value	sr^2^	VIF
		0.233	27.909	<0.0001		
**NRS Pain**	1.139			0.002	0.028	1.164
**Depression HADS**	1.608			0.000	0.090	1.234
**Paid Job**	-5.455			0.008	0.021	1.076

R^2^ = adjusted explained variance, sr^2^ = squared semi-partial correlation, VIF = variance inflation factor.

Abbreviations: NRS, Numeric Rating Scale; HADS, Hospital Anxiety and Depression Scale.

## Discussion

This study shows high prevalence rates of pain and fatigue in a large population of patients with MO. The prevalence of fatigue was significantly higher in MO patients compared with healthy subjects and patients with RA. In addition, pain and fatigue were linked and both were associated with several variables such as fear avoidance beliefs, anxiety and depression, which explained 45–46% of the variance in NRS scores.

### Pain in MO

In accordance with previous literature [6,7,15] pain was a major problem in our study participants with MO. Notably, neuropathic pain was remarkably high with a prevalence of 30% among our MO patients. To our knowledge, this observation has not yet been reported in such detail. A possible explanation for this high rate of neuropathic pain is that osteochondromas arising from the long bones can compress vessels and nerves. Another explanation could be a high incidence of neuropraxia as a complication of surgical procedures. In addition, Darilek et al. demonstrated that 55.1% of MO patients also have generalized pain throughout the body that cannot directly be traced back to the location of the osteochondromas [6]. The cause of this generalized pain is unknown but perhaps nociplastic pain and central sensitization play a more prominent role in MO. Future research should focus on the different possible pain models, particularly exploring the interplay of nociceptive, neuropathic, and nociplastic components in MO, thus elucidating the potential for a mixed-type pain phenotype. Goud et al. reported on the significant impact of pain in MO on physical and social functioning such as needing to change jobs or stop working in 28% of the patients [7]. These findings underline the complexity of the symptom pain and the multidimensional consequences for MO patients in their daily lives.

### Fatigue in MO

As suspected by our clinicians prior to this study, fatigue is a highly prevalent symptom and reported by 90.4% of the cases. To our knowledge, only one previous study has specifically addressed fatigue in MO [16]. In that study patients were recruited from a national registry of rare diseases and the Norwegian MO association. Their series of 21 adult patients showed significantly higher fatigue (i.e., 86%) compared to the general population and RA, which is in line with our findings. However, the prevalence of severe fatigue in their study was 71% and in our study population 55%. Due to the small sample size of that study and possible selection bias of participants it is difficult to generalize the finding of the study of Bathen et al.[16] to the entirety of MO population. In their study they recruited participants from the national registry of rare diseases and the Norwegian MO association. These participants might be more inclined to participate due to possible greater disease severity. Furthermore, the data was collected during a meeting of the Norwegian association with unclear conditions which could affect the responses of patients and even result in social desirability bias.

Although fatigue is a common symptom of chronic diseases and has recently gained more attention as a manifestation of long-term COVID-19, there is still no uniform definition for fatigue. Several definitions can be found in the literature [34]. Despite the absence of a clear definition, self-report measurements of fatigue have proven valuable for research [34]. In this study, the NRS was selected due to its widespread use in daily Dutch healthcare practice. Similarly, the CIS questionnaire, originally developed in the Netherlands and equipped with reference scores for the general Dutch population, was chosen for its ability to mitigate potential confounding effects of cultural differences. This approach ensures a robust framework for analyzing fatigue across its various dimensions.

In this study, the hypothesis that patients with MO experience more fatigue than healthy controls and patients with RA is confirmed. Pain and fatigue have often been analysed in the before mentioned study groups [19,32,35]. However, the question remains whether the identified associated variables for pain and fatigue in those studies can be extrapolated to MO, in other words are they disease-specific or trans-diagnostic.

### Factors associated with pain and fatigue

Using the theoretical framework of the ICF-models and based on previous literature we hypothesized that certain patient characteristics, psychosocial status and disease characteristics are significantly associated with pain and fatigue.

In this respect, the MO study population is quite interesting due to the heterogeneity of the mutation expression (EXT1-EXT2) of the disease. Several studies on genotype and phenotype argue that patients with EXT1 mutations and males are more affected by the disease [2,5,10–12] while others found no differences [13,14]. It is theorized that the later epiphyseal closure in boys prolongs the effect of EXT mutations and therefore generates more osteochondromas, greater deformities and consequently more functional limitations. Interestingly, in our study female patients, not males, reported more pain and fatigue. The existing literature on chronic pain states that men and women differ in their responses to pain, possibly because women are more sensitive in perceiving pain due to various biopsychosocial differences [36]. Whether the observed gender difference in our study is due to disease specific factors such as phenotype severity or genotype, or broader biopsychosocial aspects is unknown. Nonetheless, it is an interesting finding that female MO patients seem more affected when it comes to pain and fatigue. Future research should focus on the relationship between genotype, phenotype and clinical outcome measures such as pain and fatigue.

The main treatment of MO nowadays is surgery. The more surgical procedures have been performed, the higher the pain reported. This contra-intuitive finding is consistent with the findings of Darilek et al.[6]. In their series patients with MO who had surgery were 3.8 times more likely to report pain. The direction of causality is yet to be determined. Could it be that patient with a worse phenotype acquire more surgery and therefore it is the phenotype and not the surgery that is causing the higher reports of pain? Further research is needed to clarify this.

In contrast with the literature on chronic musculoskeletal diseases, other patient characteristics such as a higher BMI, age, lower educational levels, and comorbidities were not associated with pain or fatigue in MO [35]. Also, the malignant degeneration into chondrosarcomas and a negative family history of MO were not associated with pain and fatigue. However, patients with a paid job reported lower fatigue levels, which is consistent with literature about other chronic diseases [18,35,37]. In this regard, Goertz et al. showed that being unemployed was significantly associated with severe fatigue [35]. Additionally, Enns et al. reported that fatigue is a significant predictor of work and activity impairment in several chronic diseases such as RA [21]. Fatigue is a meaningful clinical outcome and influences patients’ day to day lives. Reduction in fatigue of 20% can result in meaningful clinical outcomes such as return to work in patients with musculoskeletal pain [18].

Other variables such as fear avoidance beliefs, catastrophizing, anxiety and depression have been found to be associated with pain and fatigue. Although the mean group scores in this study population for the HADS, PCS and FABQ were below the cut-off score for abnormality, an association was still present. This is also consistent with the current literature [35,38]. In a meta-analysis of patients with osteoarthritis a positive association between pain severity and both anxious and depressive symptoms was found (resp. r = 0.31 and r = 0.36 p<0.001) [38]. In patients with low back pain a significant association of fatigue severity with depression, anxiety and pain catastrophizing was reported [39]. Koyoma et al. reported that the expectation of pain activates the same areas of the brain that actual pain does [40]. Therefore, one can discuss that pain catastrophizing is associated with higher pain scores as is shown in this study and others [41,42]. Early screening for psychological disorders may be beneficial for the treatment of fatigue and pain, and even result in lower health costs. Prior studies among patients with chronic pain actually reported higher direct health costs in case of mental disorders [43,44]. Cognitive behavioural therapy and exercise therapy have been proven to reduce fatigue and pain in a variety of chronic diseases [45,46] and could be helpful in MO patients.

Pain and fatigue are complex symptoms and as shown in this study independently associated with each other. This finding is in accordance with several studies on different chronic disorders such as COPD, RA, and diabetes mellitus [35,47]. The exact mechanism of the experience of pain and fatigue as well as their relationship is still widely discussed in the literature. It is hypothesized that a temporal relationship exists between pain and fatigue and that physical or psychological energy required to cope with persistent pain depletes the energy resources that contribute to the experience of fatigue [48]. Manning et al. suggested that fatigue sensitivity (i.e., the reaction of patients on fatigue symptoms) may be involved in the mechanism of pain experience [39]. However, van Dartel et al. could not find a temporal relationship and stated that both should always be treated, because it cannot be expected that an improvement of pain improves fatigue and vice versa [49].

## Limitations

The data in this study was obtained using validated questionnaires and thus is based on self-report with a risk of recall bias. The reference scores of healthy subjects used to compare fatigue (CIS) date back to a Dutch study from 1994 and may be less representative nowadays. To our knowledge no updates have been published for the Dutch population. In this study it is impossible to determine the direction or causality of the variables due to its cross-sectional design. Longitudinal studies could offer a solution to this limitation in future research.

Another disadvantage of this study design is the insufficient control over potential confounding factors such as medication use, particularly painkillers, and genetic variations among patients. Although we identified that 76 patients were using some form of painkillers, the lack of detailed dosage and frequency information prevented us from analyzing potential associations with other outcome measures. Additionally, while genetic mutations were not explored as part of our study objectives, understanding the specific genotypes could provide valuable insights in future research into patient symptoms and their impact on quality of life. However, obtaining genotypic data was not feasible due to the inclusion of participants recruited from the national MO association. Furthermore, an underrepresentation of participants with mild disease severity cannot be excluded. In our study population 8.5% of the patients have had a chondrosarcoma which is higher than reported in the literature (i.e., 2–6%) [2–5]. This could mean that patients that are more affected were more likely to participate. However, to ensure enrolment of patients with a variety of disease severity we recruited former patients from the hospital database and patients at their regular check-ups in the outpatient clinic as well as via the national patient association resulting in the largest cohort of MO on fatigue. With a response rate of 90.1%, a substantial study population of 353 participants, and the rare prevalence of MO, the risk of selection bias is reduced, rendering our findings more generalizable to the entire MO population.

## Implications for clinical practice and future research

In this study pain is confirmed to be highly prevalent in MO but fatigue appears to be a major impairing symptom as well. In other studies on chronic diseases several associated variables for pain and fatigue have been reported and they matched our results [32,35,38]. However, the several associated parameters in the study of Goertz et al. only explained 26% of the explained variance in fatigue [35], whereas in our study it was 46%. The identified variables in this study should be taken into account while designing an intervention for both pain and fatigue to strive for better clinical outcomes. Furthermore, these variables can help identify patients at risk for developing clinically relevant pain and fatigue levels. We found that pain is highly associated with fear avoidance beliefs and pain catastrophizing in patients with MO, while fatigue is shown to be associated with anxiety and depression. Cognitive behavioural therapy in these cases could aid in the treatment of pain and fatigue. These results underline the need of a multidisciplinary approach in the assessment and treatment of patients with MO.

Studies on chronic pain have shown that fatigue can often be more debilitating than pain [50]. Furthermore, when pain and fatigue coincide with changes in psychological factors, consideration should be given to interdisciplinary multimodal pain treatment approaches [51]. Our findings highlight the need to address both pain and fatigue in routine orthopaedic consultations and advocate for a comprehensive biopsychosocial diagnostic and treatment approach for MO patients. More research, particularly longitudinal studies, is necessary to understand the pathways underlying the relationship between pain and fatigue. Since pain and fatigue are associated in MO, both should be addressed in care of these patients. Whether fatigue and pain are more influenced by disease specific factors rather than trans-diagnostic factors warrants more research regarding the possible association of genotype, phenotype and clinical outcomes.

## Conclusion

This study identified a significant prevalence of pain and fatigue among patients with MO, with 88% reporting pain and 90% reporting fatigue. The pain and fatigue scores of MO patients were significantly more severe compared with reference scores of healthy controls and patients with RA. An association of pain and fatigue was observed, and several other variables associated with pain and fatigue have been identified that can be used for the development of treatment plans. These findings suggest that a multidisciplinary approach in the treatment of MO is warranted, integrating psychological support and physical health management to alleviate pain and fatigue and improve overall quality of life for these patients

## Supporting information

S1 Appendix Tables univariate analysis (first step of 2-step model): Table 7: univariate analysis of the dependent variable NRS–pain. First step of 2-step model. Table 8: univariate analysis of the dependent variable NRS–fatigue. First step of 2-step model. Table 9: univariate analysis of the dependent variable Checklist Individual Strength (CIS)–fatigue. First step of 2-step model. Legend tables 7–9: Abbreviations: NRS, Numeric Rating Scale; CIS, Checklist Individual strength; DN4, Douleur Neuropathic en 4 questions; HADS, Hospital Anxiety and Depression Scale; PCS, Pain Catastrophizing Scale; FABQ, Fear-Avoidance Beliefs Questionnaire; BPAQ, Baecke Physical Activity Questionnaire; BMI, Body Mass Index.
(DOCX)
