# Supplementary material for: Pain and fatigue in adult patients with multiple osteochondromas: The Netherlands
Source: PLoS One. 2024 Jul 17;19(7):e0305640. doi: 10.1371/journal.pone.0305640 (PMC11253920; doi:10.1371/journal.pone.0305640)
Supplement: S1 Appendix — First step of 2-step model. Table 8: univariate analysis of the dependent variable NRS–fatigue. First step of 2-step model. Table 9: univariate analysis of the dependent variable Checklist Individual Strength (CIS)–fatigue. First step of 2-step model. Legend tables 7–9: Abbreviations: NRS, Numeric Rating Scale; CIS, Checklist Individual strength; DN4, Douleur Neuropathic en 4 questions; HADS, Hospital Anxiety and Depression Scale; PCS, Pain Catastrophizing Scale; FABQ, Fear-Avoidance Beliefs Questionnaire; BPAQ, Baecke Physical Activity Questionnaire; BMI, Body Mass Index. (DOCX) [file pone.0305640.s001.docx]

# **Appendix A. Tables univariate analysis (first step of 2-step model)**

## **Univariate analysis for pain – NRS**

- For the first step of the two-step analysis the purposeful selected independent variables from the ICF model (Figure 1.) were analysed using univariate analysis. Variables were selected for the second step if p ≤0.25.
- Excluded variables (p>0.25): age, illness duration, positive family history, less than 6 surgical procedures, marital status (divorced, widowed, living together, single), primary education level.

**Table 7. Dependent variable NRS – pain.**

| **Independent variables** | **Unstandardized coefficients** | **R^2^** | **F** | **P- value** |
| --- | --- | --- | --- | --- |
| NRS – fatigue | 0.528 | 0.278 | 134.855 | 0.000 |
| CIS – total score | 0.056 | 0.111 | 43.875 | 0.000 |
| CIS – fatigue | 0.122 | 0.144 | 59.140 | 0.000 |
| Pain location |  |  |  |  |
| 1-2 | 1.895 | 0.068 | 25.735 | 0.000 |
| 3-4 | 2.760 | 0.005 | 1.632 | 0.202 |
| 5-7 | 4.331 | 0.046 | 17.035 | 0.000 |
| >7 | 5.124 | 0.220 | 98.723 | 0.000 |
| DN4 | 2.411 | 0.180 | 76.967 | 0.000 |
| HADS – anxiety | 0.213 | 0.105 | 41.154 | 0.000 |
| HADS – depression | 0.279 | 0.139 | 56.672 | 0.000 |
| PCS | 0.129 | 0.222 | 100.036 | 0.000 |
| FABQ | 0.066 | 0.273 | 131.770 | 0.000 |
| BPAQ | -0.297 | 0.27 | 7.453 | 0.007 |
| Gender | 1.2 | 0.055 | 20.235 | 0.000 |
| BMI | 0.063 | 0.016 | 5.645 | 0.018 |
| Married | -0.546 | 0.017 | 1.203 | 0.186 |
| Surgical procedures |  |  |  |  |
| 6-10 | 1.108 | 0.006 | 2.275 | 0.132 |
| >10 | 1.759 | 0.047 | 17.335 | 0.000 |
| Education: Secondary level | 0.924 | 0.032 | 11.535 | 0.001 |
| Education: Tertiary level | -1.09 | 0.044 | 16.095 | 0.000 |
| Other education | 1.980 | 0.013 | 4.679 | 0.031 |
| Employment | -1.279 | 0.054 | 20.207 | 0.000 |
| Malignant degeneration | 0.995 | 0.012 | 4.140 | 0.043 |
| Comorbidity | 1.269 | 0.045 | 16.373 | 0.000 |

Abbreviations: NRS, Numeric Rating Scale; CIS, Checklist Individual strength; DN4, Douleur Neuropathic en 4 questions; HADS, Hospital Anxiety and Depression Scale; PCS, Pain Catastrophizing Scale; FABQ, Fear-Avoidance Beliefs Questionnaire; BPAQ, Baecke Physical Activity Questionnaire; BMI, Body Mass Index.

## **Univariate analysis for Fatigue – NRS**

- Excluded variables (p>0.25): age, illness duration, positive family history, less than 6 surgical procedures, marital status (divorced, widowed, single), primary education.

**Table 8. Dependent variable NRS – fatigue.**

| **Independent variables** | **Unstandardized coefficients** | **R^2^** | **F** | **P- value** |
| --- | --- | --- | --- | --- |
| Pain – NRS | 0.525 | 0.278 | 134.855 | 0.000 |
| Pain location |  |  |  |  |
| 1-2 | 1.090 | 0.072 | 27.114 | 0.009 |
| 3-4 | 2.379 | 0.003 | 26.401 | 0.000 |
| 5-7 | 3.334 | 0.032 | 11.453 | 0.000 |
| >7 | 3.820 | 0.121 | 48.374 | 0.000 |
| DN4 | 1.837 | 0.105 | 41.151 | 0.000 |
| HADS – Anxiety | 0.342 | 0.272 | 131.194 | 0.000 |
| HADS – Depression | 0.358 | 0.228 | 105.066 | 0.000 |
| PCS | 0.113 | 0.171 | 72.384 | 0.000 |
| FABQ | 0.057 | 0.211 | 93.718 | 0.000 |
| BPAQ | -0.345 | 0.033 | 8.953 | 0.003 |
| Gender | 1.443 | 0.078 | 29.611 | 0.000 |
| BMI | 0.067 | 0.18 | 6.406 | 0.120 |
| Married | -0.869 | 0.008 | 2.818 | 0.035 |
| Living together | -1.070 | 0.006 | 2.031 | 0.033 |
| Employment | -1.112 | 0.041 | 15.151 | 0.000 |
| Education: Secondary level | 0.658 | 0.016 | 5.779 | 0.017 |
| Education: Tertiary level | -0.742 | 0.020 | 7.251 | 0.007 |
| Other education | 1.064 | 0.004 | 1.346 | 0.247 |
| Surgical procedures |  |  |  |  |
| 6-10 | 0.823 | 0.005 | 1.877 | 0.029 |
| >10 | 0.944 | 0.010 | 3.468 | 0.012 |
| Malignant degeneration | 0.818 | 0.008 | 2.803 | 0.095 |
| Comorbidity | 1.407 | 0.055 | 20.468 | 0.000 |

Abbreviations: NRS, Numeric Rating Scale; DN4, Douleur Neuropathic en 4 questions; HADS, Hospital Anxiety and Depression Scale; PCS, Pain Catastrophizing Scale; FABQ, Fear-Avoidance Beliefs Questionnaire; BPAQ, Baecke Physical Activity Questionnaire; BMI, Body Mass Index.

## **Univariate analysis for Fatigue - Total CIS**

- Excluded variables (p>0.25): illness duration, positive family history, malignant degeneration, primary and other education, marital status (married, widowed, living together, single), less than 3 pain locations, less than 11 surgical procedures.

**Table 9. Dependent variable CIS – total score.**

| **Independent variables** | **Unstandardized coefficients** | **R^2^** | **F** | **P- value** |
| --- | --- | --- | --- | --- |
| Pain – NRS | 1.987 | 0.111 | 43.875 | 0.000 |
| Pain location |  |  |  |  |
| 3-4 | 3.429 | 0.005 | 1.845 | 0.175 |
| 5-7 | 7.577 | 0.003 | 1.225 | 0.011 |
| >7 | 11.616 | 0.055 | 20.458 | 0.000 |
| DN4 | 6.227 | 0.034 | 12.261 | 0.001 |
| HADS – Anxiety | 1.194 | 0.093 | 35.955 | 0.000 |
| HADS – Depression | 2.026 | 0.207 | 91.548 | 0.000 |
| PCS | 0.516 | 0.099 | 38.617 | 0.000 |
| FABQ | 0.236 | 0.100 | 38.935 | 0.000 |
| BPAQ | -2.526 | 0.049 | 13.737 | 0.000 |
| Gender | 3.675 | 0.014 | 5.025 | 0.026 |
| Age | 0.071 | 0.006 | 1.989 | 0.159 |
| BMI | 0.536 | 0.033 | 11.794 | 0.001 |
| Marital status - Divorced | 7.543 | 0.009 | 3.045 | 0.082 |
| Employment | -9.471 | 0.084 | 32.171 | 0.000 |
| Education:  Secondary level | 4.968 | 0.026 | 9.322 | 0.002 |
| Education: Tertiary level | -5.115 | 0.027 | 9.716 | 0.002 |
| >10 surgical procedures | 4.744 | 0.015 | 1.823 | 0.036 |
| Comorbidity | 6.057 | 0.029 | 10.328 | 0.001 |

Abbreviations: NRS, Numeric Rating Scale; DN4, Douleur Neuropathic en 4 questions; HADS, Hospital Anxiety and Depression Scale; PCS, Pain Catastrophizing Scale; FABQ, Fear-Avoidance Beliefs Questionnaire; BPAQ, Baecke Physical Activity Questionnaire; BMI, Body Mass Index.
